# Definition und Behandlung der A.-mesenterica-superior-Revaskularisations- und -Dissektions-assoziierten Diarrhö (SMARD-Syndrom) in Deutschland

**DOI:** 10.1007/s00104-021-01427-4

**Published:** 2021-06-08

**Authors:** Patrick Téoule, Katharina Tombers, Mohammad Rahbari, Flavius Sandra-Petrescu, Michael Keese, Nuh N. Rahbari, Christoph Reißfelder, Felix Rückert

**Affiliations:** 1 Chirurgische Klinik, Universitätsmedizin Mannheim, Medizinische Fakultät Mannheim, Universität Heidelberg, Theodor-Kutzer-Ufer 1–3, 68167 Mannheim, Deutschland; 2 Chirurgische Klinik, European Center for AngioScience (ECAS) Universitätsmedizin Mannheim, Medizinische Fakultät Mannheim, Universität Heidelberg, Mannheim, Deutschland

**Keywords:** Pankreas, Pankreatoduodenektomie, Pankreatektomie, Ernährungsberatung, Morbidität, Pancreas, Pancreatoduodenectomy, Pancreatectomy, Nutrition counseling, Morbidity

## Abstract

**Hintergrund:**

Die A. mesenterica superior (AMS) wird im Rahmen von Pankreasresektionen (PR) und mesenterialen Gefäßeingriffen (MG) freigelegt und disseziert. Eine dadurch entstandene Schädigung des umliegenden ex- und intrinsischen vegetativen Nervenplexus kann zu einer passageren oder therapierefraktären Diarrhö führen.

**Fragestellung:**

Die vorliegende Studie soll einen Überblick über den derzeitigen Stellenwert der AMS-Revaskularisations- und -Dissektions-assoziierten Diarrhö („superior mesenteric artery revascularisation and dissection-associated diarrhea“[SMARD]-Syndrom) in Deutschland geben.

**Material und Methoden:**

Nach selektiver Literaturrecherche (SLR) mit der Fragestellung, ob und wie häufig eine postoperativ neu aufgetretene Diarrhö nach PR und MG vorkommt, wurde eine Onlineumfrage versendet.

**Ergebnisse:**

Die SLR (*n* = 4) bestätigte, dass eine postoperativ neu aufgetretene Diarrhö eine häufige Komplikation nach Präparation zur Revaskularisation (RV) bzw. Dissektion (DIS) der AMS ist (Inzidenz ca. 62 %). Therapierefraktäre Verläufe sind selten 14 %. 54 von 159 Zentren beteiligten sich an der Umfrage. 63 % gaben an, eine AMS-RV/-DIS im Rahmen von PR oder MG durchzuführen. Der Durchschnitt an PR pro Zentrum lag 2018 bei 47 und bei 49 im Jahr 2019. Fünf MG erfolgten durchschnittlich in beiden Jahren pro Zentrum. Drei Patienten litten durchschnittlich am SMARD-Syndrom.

**Diskussion:**

Diese Umfrage erfasst erstmals den derzeitigen Stellenwert des SMARD-Syndroms in Deutschland. Bisher fehlen Empfehlungen zur Therapie einer solchen Diarrhö. Die Ergebnisse zeigen, dass zunächst eine symptomatische Therapie erfolgen sollte. Aufgrund der Komplexität der Pathophysiologie sind kausale Therapieansätze bislang nicht entwickelt.

## Abstract (en)

**Background:**

The superior mesenteric artery (SMA) is exposed and dissected during pancreatic resections (PR) and mesenteric vascular surgery (MVS). The resulting damage of the surrounding extrinsic and intrinsic vegetative nerve plexus can lead to a temporary or treatment refractory diarrhea.

**Objective:**

This study aimed to provide an overview of the current status of SMA revascularization and dissection-associated diarrhea (SMARD syndrome) in Germany.

**Material and methods:**

After a selective literature search (SLS) on the frequency of newly developed postoperative diarrhea after PR and MVS, an online survey was initiated.

**Results:**

The SLS (*n* = 4) confirmed that newly developed postoperative diarrhea is a frequent complication after preparation for revascularization (RV) or dissection (DIS) of the SMA (incidence approximately 62%). Treatment refractive courses were relatively uncommon with 14%. Out of 159 centers 54 took part in the survey and 63% stated that they carried out an SMA RV/DIS during PR or MVS. The average PR per center was 47 in 2018 and 49 in 2019. The average MVS was 5 per center in both years and on average 3 patients suffered from SMARD syndrome.

**Conclusion:**

This survey recorded the current status of the SMARD syndrome in Germany for the first time. So far there are no recommendations for the treatment of such a diarrhea. The results show that initially a symptomatic treatment should be carried out. Due to the complexity of the pathophysiology, causal treatment approaches have not yet been developed.

Die A. mesenterica superior (AMS) wird im Rahmen von Pankreasresektionen (PR) und mesenterialen Gefäßeingriffen (MG) immer häufiger freigelegt. Dadurch kann der umliegende vegetative Nervenplexus schwer und irreversibel geschädigt werden. Die fehlende oder beeinträchtigte neurale Innervation führt zu einer passageren, teils aber auch therapierefraktären Diarrhö. Bisher gibt es für dieses Krankheitsbild keinen Namen. Im Rahmen der Studie möchten wir das von uns als „SMARD“ („Superior Mesenteric Artery Revascularisation and Dissection-associated Diarrhea“) bezeichnete Syndrom näher beleuchten. Der Fokus der Arbeit liegt vornehmlich auf der Pankreaschirurgie.

## Hintergrund und Fragestellung

Die Innervation des Dünndarms (DD) lässt sich in einen ex- und intrinsischen Nervenplexus unterteilen[1]. Die extrinsische Kontrolle erfolgt über den Plexus mesentericus superior (PMS), der aus autonomen Nerven (postganglionäre sympathische und präganglionäre parasympathische Nervenfasern) besteht, die der AMS und ihren Ästen zum DD und rechten sowie transversalen Dickdarm folgen [2]. Das enterische Nervensystem (ENS) bzw. der intrinsische Nervenplexus bestehen aus dem vollständig intramural lokalisierten myenterischen und submukosalen Plexus (Auerbach und Meissner; [1]). Obwohl das ENS unabhängig agiert, kann die Stimulation durch das parasympathische und sympathische System die gastrointestinalen (GI) Funktionen verbessern und/oder hemmen [1, 3, 4]. Darüber hinaus sind diverse Hormone (Serotonin, Ghrelin, Motilin) an der Regulation der GI-Sekretion und -Motilität beteiligt.

**Figure 1:**
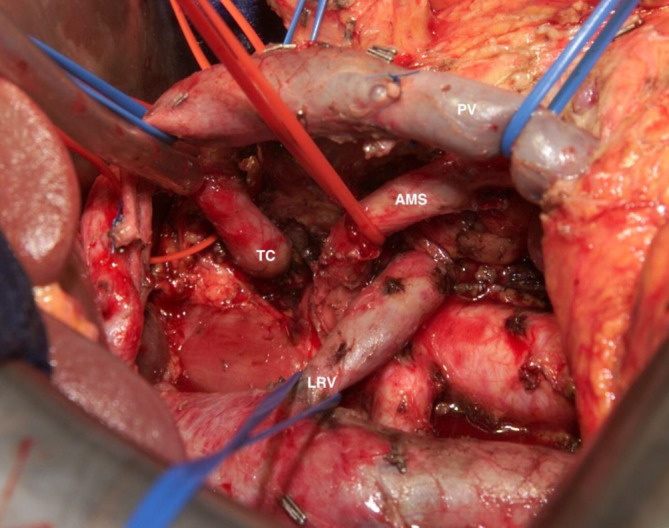

Vor diesem Hintergrund wird ersichtlich, dass es im Rahmen ausgedehnter PR (Abb. 1) und MG zur Denervierung des DD durch Schädigung des ex- und intrinsischen Plexus kommen kann [5]. Daraus können postoperative Sekretions- und Motilitätsstörungen bzw. eine therapierefraktäre Diarrhö und sekundär eine Malnutrition der Patienten resultieren [6]. Sofern die Diarrhö therapeutisch schwer beherrschbar oder therapierefraktär ist, kommt es zu einer erheblichen Minderung der Lebensqualität (LQ; [7–10]). Es besteht zudem das Risiko, dass eine empfohlene adjuvante Chemotherapie nicht durchgeführt werden kann, da sie die Diarrhö aggravieren könnte [11, 12]. Eine weitere Form der postoperativen Diarrhö nach PR ist eine osmotische Diarrhö durch exokrine Pankreasinsuffizienz (PI). Deshalb wurde eine exokrine PI als Ausschlusskriterium in die Definition des SMARD-Syndroms aufgenommen. Der Ausschluss einer exokrinen PI wurde durch das fehlende Ansprechen auf Enzymgabe definiert.

Der Symptomkomplex wurde unserer Kenntnis nach bis dato kaum beschrieben. Demzufolge sehen wir es als notwendig an, tiefere Erkenntnisse in Bezug auf Inzidenz und Behandlung zu erlangen, um in der Zukunft eine bessere Erforschung zu ermöglichen und von Diarrhöen anderer Ursache abzugrenzen.

## Studiendesign und Untersuchungsmethoden

Die vorliegende deskriptive, anonymisierte, deutschlandweite via E‑Mail versendete Umfrage, adressiert an alle deutschen Unikliniken und zertifizierten Pankreas- sowie Gefäßzentren, soll die Anzahl von AMS-Revaskularisationen (RV) und/oder Dissektionen (DIS) sowie den derzeitigen Stellenwert des SMARD-Syndroms in Deutschland und den therapeutischen Umgang damit erfragen. Grundlage war die erstmalige Definition des Syndroms. Eine folgende selektive Literaturrecherche (SLR) in den Datenbanken ©PubMed und ©Cochrane Library erfasst die bisherige Datenlage des Symptomkomplexes. Darüber hinaus soll die Studie die nach Meinung der Autoren dringend notwendige Diskussion über das SMARD-Syndrom in der Chirurgie anstoßen.

### Literaturrecherche

Die SLR (10. bis 12.05.2019) umfasste folgende Suchbegriffe: diarrhea, arteria mesenterica, arterial resection, pancreatectomy und enteric nervous system. Sie wurde gemäß den Richtlinien für Berichterstattungselemente für systematische Überprüfungen und Metaanalysen (PRISMA) durchgeführt [13]. Studien vor dem Jahr 2000, in nicht englischer oder deutscher Sprache und Truncus-coeliacus-Resektionen wurden ausgeschlossen. Diarrhö musste als Endpunkt aufgeführt sein. Die Ergebnisse wurden unabhängig voneinander von zwei Autoren (KT und FR) geprüft und anhand der PRISMA-Leitlinien selektioniert. Bei Unstimmigkeiten wurde ein dritter Autor (PT) konsultiert.

### Definition

Die Definition des SMARD-Syndroms erfolgt erstmals im Rahmen dieser Arbeit und wird von uns wie folgt vorgeschlagen:

1. neu aufgetretene, postoperativ persistierende medikamentös therapiebedürftige Diarrhö > 4 Wochen nach AMS-Revaskularisation oder -Dissektion *und*
2. Ausschluss einer exokrinen Pankreasinsuffizienz im Stuhl (d. h. keine Besserung der Symptomatik durch adäquate Substitution von Pankreasenzymen zu den Mahlzeiten) und/oder Ausschluss einer antibiotikainduzierten Diarrhö.

Als Diarrhö wurde dabei die Definition der WHO verwendet: eine Stuhlentleerung > 3 mal pro Tag von regelrechter Konsistenz oder verminderte oder flüssige Stuhlkonsistenz oder häufigere Stuhlentleerung als üblich für die jeweilige Person [14].

Anzumerken ist, dass sich das hier definierte SMARD-Syndrom ausschließlich auf eine Schädigung des PMS bezieht, welcher die AMS umgibt. Davon abzugrenzen ist eine Diarrhö, die auf eine Schädigung des kranial gelegenen Plexus coeliacus zurückzuführen ist, wie z. B. im Rahmen einer Appleby-Operation, bei der der Truncus coeliacus aufgrund einer Infiltration reseziert wird.

### Onlineumfrage und Statistik

**Figure 2:**
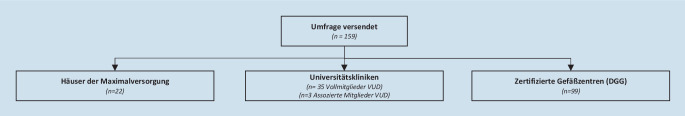

Einen Überblick über die Verteilung der versendeten Umfrage gibt Abb. 2. Die entsprechenden Abteilungen wurden berücksichtigt, wenn sie auf der Homepage des ©Verbandes der Universitätsklinika Deutschlands e. V. aufgeführt waren. Zertifizierte Pankreas- und Gefäßzentren wurden berücksichtigt, wenn sie von der Deutschen Gesellschaft für Allgemein- und Viszeralchirurgie (©DGAV) als Studien‑, Dokumentations‑, Qualitätszentrum (©StuDoQ) bzw. durch die Deutsche Gesellschaft für Gefäßchirurgie und Gefäßmedizin (©DGG) auf der entsprechenden Homepage im November 2019 als solches ausgewiesen wurden. Elf Fragen (offen und geschlossen) mit einem offenen Kommentarfeld wurden versendet (Tab. 1**)**. Um den aktuellen Stand erfassen zu können, wurden lediglich die Jahre 2018/2019 berücksichtigt. Antworten bis zum 31.01.2020 wurden in die Studie eingeschlossen.

Als statistische Mittel wurden aufgrund der deskriptiven Fragen die Werte in absoluten und relativen Häufigkeiten (Standardabweichung [SD] inklusive Spannweite [Range] und der Median inklusive Interquartilsabstand [IQR]) angegeben. Zum Vergleich beider Gruppen wurde der nichtparametrische Test nach Mann-Whitney‑U für den Median bzw. der T‑Test für Mittelwerte verwendet, um bei *p* ≤ 0,05 mögliche signifikante Unterschiede aufzuzeigen.

Als Umfragetool wurde die Umfragesoftware ©SurveyMonkey (One Curiosity Way, San Mateo, CA, USA) genutzt und im Januar 2020 wurde die Umfrage durch den Erst- und Letztautor an die auf der Homepage verfügbaren Adressen der jeweiligen Klinikdirektoren und/oder Abteilungsleiter versendet. Die Definition des SMARD-Syndroms wurde genannt, sodass eine einheitliche Informationsbasis geschaffen wurde. Die eingegangenen Antwortbögen wurden fortlaufend durchnummeriert, ohne dass ein Rückschluss auf den Absender möglich war.

**Table 1:** 

Nr	Frage	Antwortauswahl	Antwortrate
1	Führen Sie im Rahmen pankreas- oder gefäßchirurgischer Eingriffe eine Resektionen und Rekonstruktion der AMS durch?	Ja	100
		Nein	
2	Wie viele pankreaschirurgische Eingriffe führten Sie (in Ihrem Zentrum) in den letzten beiden Jahren durch?	2018	35
		2019	
3	Wie viele gefäßchirurgische Eingriffe mit Resektion oder Dissektion der AMS führten Sie (in Ihrem Zentrum) in den letzten beiden Jahren durch?	2018	35
		2019	
4	Wie oft führten Sie eine Revaskularisation oder Dissektion der AMS jährlich durch?	2018	28
		2019	
5	Wie viele dieser Patienten entwickelten eine neu aufgetretene Diarrhö?	2018	30
		2019	
6	Wurden die Patienten daraufhin (bez. SMARD-Syndrom) chirurgisch, internistisch oder interdisziplinär behandelt?	Chirurgisch	22
		Internistisch	
		Interdisziplinär	
7	Wie therapier(t)en Sie das SMARD-Syndrom?	Freitext	17
8	Wurde planmäßig ein Follow-up Ihrer Patienten mit SMARD-Syndrom durchgeführt?	Ja	20
		Nein	
9	Wie viele Patienten sprachen auf die Therapie des SMARD-Syndroms an?	Freitext	17
10	Bitte nennen Sie den Anteil an Respondern in Abhängigkeit der Art des Eingriffes:	Pankreaschirurgisch	17
		Gefäßchirurgisch	
11	Wie gehen Sie bei Nonrespondern vor?	Freitext	17

*Antwortrate*: Anzahl der erhaltenen Antworten von der Gesamtzahl der Teilnehmer (*n* = 54) in Prozent

*AMS* Arteria mesenterica superior, *SMARD* „superior mesenteric artery revascularisation and dissection-associated diarrhea“

## Ergebnisse

**Figure 3:**
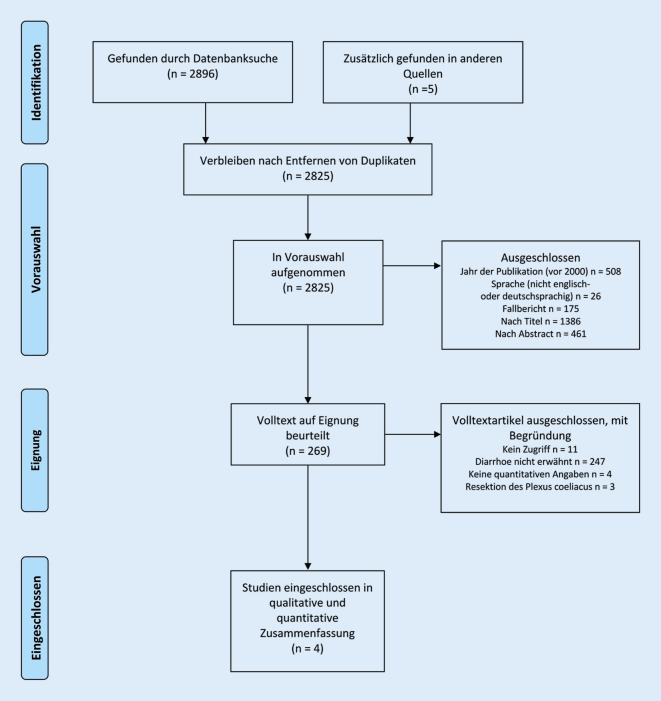

In die SLR wurden vier Studien eingeschlossen, die eine postoperativ neu aufgetretene Diarrhö beschreiben (Tab. 2; [11, 15–17]). Einen Überblick über die verschiedenen Phasen der selektiven Literaturrecherche gibt das in Anlehnung an das PRISMA-Flussdiagramm erstellte Diagramm (Abb. 3). In der Arbeit von Settmacher et al. werden die Erfahrungen mit Viszeralarterienrekonstruktionen mittels Homografts bei Pankreasresektionen dargestellt [17]. Nimura et al. (*n* = 101) und Inoue et al. (*n* = 233) stellen die Ergebnisse in Bezug auf das Ausmaß der Lymphadenektomie (Standard vs. erweitert) nach Pankreatoduodenektomie (PD) gegenüber [11, 15]. Mizuno et al. (*n* = 25) beschreiben ein Operationsverfahren zur Dissektion der AMS bzw. des PMS im Rahmen von PD [16].

Insgesamt wurden 1399 Patienten mit PR beschrieben, davon 220 (16 %) mit Resektion oder Dissektion der AMS. Postoperativ trat bei 137 (62 %) Patienten eine Diarrhö auf. Kumulativ war die Diarrhö in 19 (14 %) Fällen therapierefraktär [11, 15, 17]. Zwei Studien nannten Therapien [15, 16]. Zwei (1 %) Patienten erhielten Loperamid [16] ohne konkrete Dosisangaben. Alternativ wurde bei 22 (16 %) Betroffenen Tanninalbuminat (3–6 g/Tag) oder natürliches Aluminiumsilikat (3–6 g/Tag; [15]) und bei 70 (51 %) Patienten Opiumtinktur verabreicht (0,2–7,2 ml/Tag; [15]).

**Table 2:** 

Autor	Jahr	Anzahl	Anzahl AMS-Resektion/Dissektion (%)	Chirurgisches Vorgehen (*n*)	Anzahl symptomatischer Patienten (Diarrhö) (%)	Anzahl therapierefraktärer Patienten (%)	Therapievorschlag
Settmacher et al. [17]	2004	1040	7 (1)	Pankreatektomie (5)/Whipple (2) arterielle Rekonstruktion mit Homograft/direkte Rekonstruktion	1 (14)	1 (14)	–
Nimura et al. [11]	2012	101	50 (50)	PD (11)/PPPD (23)/SSPPD (16) + erw. LAD^a^	42 (84)	12 (29)	–
Mizuno et al. [16]	2014	25	5 (20)	„Anterior-approach PD“ + „Hanging“-Manöver	2 (40)	Keine Angabe	+
Inoue et al. [15]	2018	233	158 (68)	„Artery-first approach PD“ + LAD LV3 (117) oder „extended LV3“^b^ (41)	92 (58)	6 (5)	+

*AMS *Arteria mesenterica superior,* Homograft* entweder kryokonservierte Beckenarterie oder AMS,* LAD* Lymphadenektomie, *LV* Level, *erw.* erweitert, *PD* Pankreatoduodenektomie, *PMS* Plexus mesentericus superior, *PPPD* pyloruserhaltende Pankreatoduodenektomie, *SSPPD* subtotale magen(„stomach“)erhaltende Pankreatoduodenektomie, *+* ja, *−* nein

^a^In der Tabelle werden die Anzahl der Eingriffe der AMS in Relation zur Gesamtanzahl der Patienten und die Inzidenz einer neu aufgetretenen postoperativen Diarrhö dargestellt. Sofern keine Rekonstruktion aufgeführt ist, wurde keine durchgeführt. Alle aufgeführten Artikel sind Originalarbeiten

^b^Nach der Japan Pancreas Society. General Rules for the Study of Pancreatic Cancer [18]

### Ergebnisse der Umfrage

Insgesamt nahmen 34 % (54/159) der Zentren an der Umfrage teil. 34 Teilnehmer (63 %) bejahten, an ihrem Zentrum PR oder MG mit AMS-Beteiligung durchzuführen. 19 Zentren nannten die Anzahl der PR und MG der beiden letzten Jahre (Tab. 3). 15 Teilnehmer (28 %) gaben die Häufigkeit der pro Jahr durchgeführten PR und MG an.

**Figure 4:**
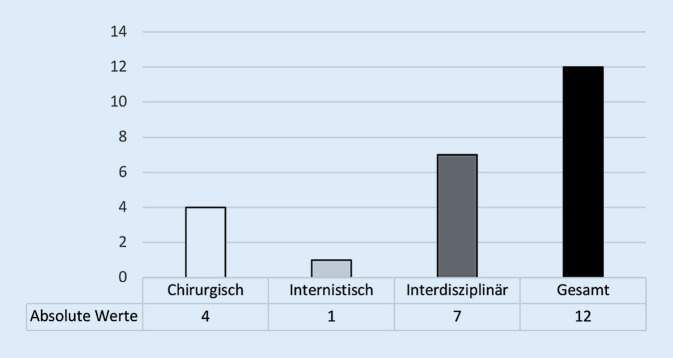

**Figure 5:**
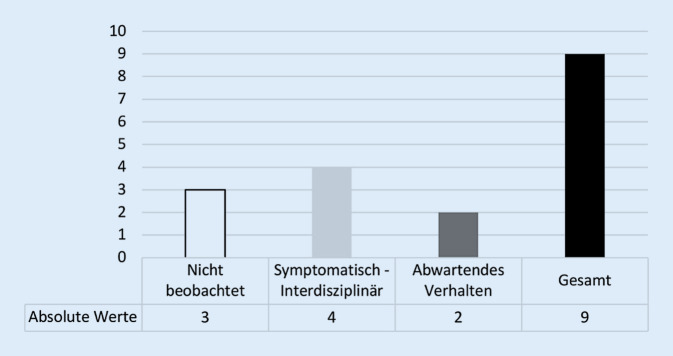

Mit knapp 60 % erfolgte die Therapie des SMARD-Syndroms am häufigsten interdisziplinär (Abb. 4) und mittels antipropulsiver Medikation in Kombination mit Flüssigkeitssubstitution (67 %). Neun Zentren (17 %) machten eine Angabe über die Therapieansprechrate, wobei in 5 Fällen eine Zahlenauswertung möglich war. Die Breite der angegebenen absoluten Responderrate lag zwischen 50 und 95 % und unterschied sich nicht zwischen den Eingriffsarten (MG: 17–95,0 % vs. PR: 50–95 %; *p* = 0,85). Das Vorgehen bei Nonrespondern wurde von 9 Zentren (17 %) dargelegt (Abb. 5). Das Kommentarfeld nutzen 8 Teilnehmer (15 %; Tab. 4). Eine Übersicht über die Anzahl der eingegangenen Antworten gibt Tab. 1.

**Table 3:** 

Nummer	Frage	2018	2019	*p*-Wert
2	*Anzahl Pankreasresektionen/Jahr*			
	Durchschnitt (SD; Range)	47,2 (39,4; 0–120)	49,4 (40,3; 0–120)	–
	Median (IQR)	40 (6–72)	45 (6–76)	0,78
3	*Anzahl Gefäßrevaskularisationen/Jahr*			
	Durchschnitt (SD; Range)	5,0 (6,8; 0–30)	5,1 (7,0; 0–30)	–
	Median (IQR)	2 (2–6)	3 (1–6)	0,90
4	*Anzahl AMS-Resektion oder Dissektion/Jahr*			
	Durchschnitt (SD; Range)	6,8 (12,50; 1–50)	6,9 (12,40; 0–50)	–
	Median (IQR)	2 (2–6)	3 (2–6)	0,83
5	*Anzahl Patienten mit neu aufgetretener Diarrhö*			
	Durchschnitt (SD; Range)	3,3 (7,7; 0–30)	3,3 (7,7; 0–30)	–
	Median (IQR)	0 (0–4)	0 (0–4)	0,98

Zum Vergleich beider Gruppen wurde der nichtparametrische Test nach *Mann-Whitney‑U *zur Untersuchung zweier unabhängiger Stichproben verwendet

*AMS* A. mesenterica superior, *SD* „standard deviation“, *IQR* „interquartile range“

**Table 4:** 

Teilnehmer	Antwort
A	Die Resektion erfolgt bei uns nur im absoluten Notfall. Die Dissektion erfolgt beim Pankreaskarzinom nicht zirkulär, sondern nur auf der rechten Hemizirkumferenz
B	Schätzwerte!!
C	Bislang kein eindeutiges SMARD-Syndrom erlebt, meist exokrine Insuffizienz Hauptursache
D	Wir kennen das klinische Problem nicht
E	Bisher kein SMARD-Syndrom realisiert, interessante Fragestellung!
F	Sehr selten, nicht vorhersehbar
G	Alle Zahlen sind Schätzungen aus unserer klinischen Erfahrung
H	Relevantes Problem, bisher auch in unserem Zentrum wahrscheinlich zu wenig im Langzeitverlauf begleitet. Oft natürlich relativ kurzes Follow-up bei ausgeprägten Pankreaskarzinomen (Letalität)

*SMARD* „superior mesenteric artery revascularisation and dissection-associated diarrhea“

## Diskussion

Diese Umfrage stellt die erste Evaluation bezüglich der Inzidenz, Dokumentation und Therapie einer postoperativ neu aufgetretenen Diarrhö nach chirurgischen Eingriffen im Bereich der AMS in Deutschland dar. Bisher gibt es keine Hinweise in der aktuellen S3-Leitlinie des Pankreaskarzinoms bzw. der Deutschen Gesellschaft für Ernährung zum Auftreten und zur Therapie einer solchen Diarrhö sowie zur Erfassung der LQ betroffener Patienten [19, 20]. Die Ergebnisse der Umfrage und der SLR zeigen jedoch, dass eine solche Diarrhö eine durchaus häufige und relevante Komplikation darstellt. Die SLR ergab, dass die Inzidenz einer neu aufgetretenen postoperativen Diarrhö bei etwa 62 % liegt, mit knapp 14 % therapierefraktären Verläufen [11, 15–17]. Die Rücklaufquote des Surveys lässt nur eingeschränkte Aussagen zu, trotzdem spiegeln die erhobenen Zahlen den Trend wider. Sowohl die Zahlen der PR und MG allgemein als auch die aufgetretenen Fälle mit SMARD-Syndrom haben im Jahr 2019 gegenüber dem Vorjahr leicht zugenommen. Unsere Daten zeigen, dass in den letzten beiden Jahren etwa 44 % der Patienten der an der Umfrage beteiligten Zentren nach AMS-RV oder -DIS von einer postoperativen therapierefraktären Diarrhö betroffen waren. Diese Zahlen vergegenwärtigen, dass das SMARD-Syndrom eine Operationsfolge ist, die durchaus in der Klinik beobachtet wird.

Bis heute stellt die chirurgische Resektion die einzig kurative Therapie eines duktalen Pankreaskarzinoms (PDAC) dar. Bei lokal fortgeschrittenen Karzinomen kann es zu Infiltrationen des umliegenden Gewebes kommen, was die Resektabilität stark einschränken kann. Im Rahmen der operativen Exploration sollte bei Verdacht auf eine arterielle Gefäßinfiltration der erste Schritt das sog. „Artery-first“-Manöver (AFM) sein. Dadurch kann sowohl die arterielle Gefäßinfiltration selbst als auch das Ausmaß der erforderlichen Resektion und Rekonstruktion bewertet werden [21, 22]. Dabei sollte besonderer Fokus auf die AMS gelegt werden, deren technische Resektabilität durch verschiedene Herangehensweisen, bevor irreversible Schritte ausgeführt werden, geprüft werden kann. Zunächst unterscheidet man abhängig von der Lage der vermuteten Infiltration beim AFM die Dissektion der peripankreatischen Arterien von der linken oder rechten unteren Mesenterialseite sowie eine infrakolische oder suprakolische Richtung [22]. Aus diesem Grund kann die Verwendung der Terminologie des AFM nicht als einzelner chirurgischer Schritt betrachtet werden, sondern als Strategie zur Prüfung der Tumorinfiltration der AMS. Aus technischer Sicht gibt es verschiedene Möglichkeiten für die arterielle Rekonstruktion nach Resektion der AMS, einschließlich direkter Reanastomisierung (End-zu-End bis zu einem Defekt von ca. 1,5 cm), Insertion von Allotransplantaten und Ersatz durch autologe Überbrückung oder Interposition (z. B. Vena saphena) [23, 24]. Das National Comprehensive Cancer Network definiert die Resektabilität je nach betroffenem Gefäß und Ausmaß der Infiltration und rät, ebenso wie die aktuelle S3-Leitlinie, von einer Resektion der AMS ab, wenn mehr als 180° durch den Primärtumor infiltriert sind [19, 25]. Zudem wird aktuell die Dissektion von mehr als 15 Lymphknoten empfohlen [25, 26], unter anderem im Bereich der rechten Hemizirkumferenz der AMS [19].

Die Grenzen der Resektabilität und das Ausmaß der Eingriffe werden zunehmend ausgeweitet [24, 27–31]. Dies liegt zum einen an der zunehmenden Anzahl spezialisierter Zentren, was zur Senkung der Morbidität und Mortalität führt [32], und zum anderen an neuen neoadjuvant-interdisziplinären Therapieansätzen, die auch bei lokal fortgeschrittenen PDAC R0-Resektionen ermöglichen [33, 34]. Demzufolge wird sich die Anzahl der Eingriffe mit zirkulären AMS-Dissektionen und -Revaskularisationen in Zukunft mutmaßlich erhöhen, wodurch auch mit steigenden relativen Zahlen von Betroffenen mit SMARD-Syndrom gerechnet werden sollte.

Obwohl das SMARD-Syndrom keine Einschränkung der Prognose bedeutet, sollte bedacht werden, dass eine chronische, eventuell therapierefraktäre Diarrhö ein für die Patienten sehr belastendes Symptom ist und zu einer starken Minderung der LQ führen kann [7–10]. Dies belastet das Arzt-Patienten-Verhältnis, vor allem da eine etablierte Therapie nicht vorliegt.

Grundsätzlich zeigen die Ergebnisse der Umfrage und SLR sowie unsere eigenen Erfahrungen, dass zunächst eine herkömmliche symptomatische Therapie der Diarrhö angebracht ist. Essenziell ist dabei der Volumen- und Elektrolytausgleich. Die in der Literatur angegebene Therapie war zumeist Tanninalbuminat/Aluminiumsilikat bei milden bzw. moderaten Verlaufsformen [15]. Opiumtinktur und Loperamidhydrochlorid wurden in schwereren Fällen eingesetzt [15, 16]. Die Ergebnisse der Umfrage zeigten zudem, dass vor allem Opiate, aber auch Flohsamen und Apfelpektin eingesetzt werden.

Mögliche alternative Therapieansätze werden bisher vor allem in der Therapie der akuten Diarrhö oder des Reizdarmsyndroms eingesetzt. Da die Symptomatik durch dissezierte sympathische Fasern hervorgerufen wird, ist ein neuer Ansatz, die Diarrhö mit Sympathomimetika oder Parasympatholytika zu behandeln. Postsynaptische adrenerge (α und β) Rezeptoren entscheiden über die Wirkung auf die Darmmotilität. Es gibt Hinweise, dass β3- und 2‑ und α1-Agonisten inhibierend auf die glatte Muskulatur des Darmes wirken, ohne dabei durch das ENS beeinflusst zu werden [35]. Ein weiterer Wirkstoff ist der α2-Agonist Clonidin, der ebenfalls hemmend auf die Darmmotilität wirkt [36, 37]. Das parasympathische System wirkt am Magen-Darm-Trakt über muskarinerge Acetylcholinrezeptoren M1‑3 und führt bei Stimulation zu einer Kontraktion. Hier zeigten selektive M3-Antagonisten wie Zamifenacin positive Ergebnisse, da sie anticholinergische Nebenwirkungen verringern und die Darmmotilität senken [36]. Eine Alternative wären zukünftig eventuell κ‑Rezeptor-Agonisten wie Asimadolin oder Eluxadolin, die wie Loperamid zu der Klasse der Opioide gehören und eine eingeschränkte systemische Verfügbarkeit aufweisen [36, 37]. Die Limitation besteht in der Auswahl der Wirkstoffe, da diese möglichst nur peripher wirksam und nicht zentral sedierend sein sollten. Eine weitere Möglichkeit könnte Racecadotril, ein Enkephalinasehemmer, sein [37, 38]. Enkephaline binden an δ‑Rezeptoren der Enterozyten und bewirken eine direkte Senkung der cAMP-Spiegel, was eine verminderte Sekretion von Wasser zur Folge hat. Durch eine Hemmung der Enkephalinase wird die Wirkdauer der Enkephaline verlängert [36–38]. Tab. 5 gibt einen Überblick über eine mögliche medikamentöse orale Therapie.

**Table 5:** 

Präparat	Basiseinheit	Applikationszeitpunkt	Dosierung/Tag	Hinweis
*Flohsamen*				
©Mucofalk (Dr. Falk Pharma GmbH, Freiburg, Deutschland)	1 Btl. = 5 g	1 Btl. ca. 30 min vor der Mahlzeit	Sukzessive steigern bis max. 2‑2‑2	Max. Effekt zum Teil erst nach ca. 2–3 Tagen; keine Kombination der Präparate
©Agiocur (MEDA Pharma GmbH & Co. KG, Bad Homburg, Deutschland)	1 Messlöffel ~ 5 g Granulat	1 Messlöffel ca. 30 min vor der Mahlzeit	Sukzessive steigern bis max. 2‑2‑2	
©Metamucil (Procter & Gamble Service GmbH, Schwalbach am Taunus, Deutschland)	1 Btl. = 5,8 g	1 Btl. ca. 30 min vor der Mahlzeit	Sukzessive steigern bis max. 2‑2‑2	
*Opiate*				
Loperamid sublingual	2 mg	Initial 2 Schmelztabletten, dann nach jedem ungeformtem Stuhl	Sukzessive steigern bis max. 2‑2‑2	Keine Kombination der Präparate und ggf. weiter titrierend
Tinctura opii	0,9 mg	Initial 0,9 mg, dann nach jedem ungeformtem Stuhl	Sukzessive steigern	
*Elektrolyte*				
©Elotrans (STADA Consumer Health Deutschland GmbH, Bad Vilbel, Deutschland)	1 Btl	1–2 Btl. nach jedem Stuhlgang	Sukzessive steigern je nach Bedarf	–

*Btl.* Beutel, *SMARD* „superior mesenteric artery revascularisation and dissection-associated diarrhea“

Die vorliegende Arbeit hat zu berücksichtigende Limitationen. Zum einen ist die Rücklaufquote mit knapp 34 % relativ gering. Zum anderen erfolgte bedingt durch die anonyme Umfrage kein strukturiertes Follow-up mit Erinnerungsnachrichten, was die Rücklaufquote mutmaßlich erhöht hätte. Zudem erfolgte die Befragung rein deskriptiv mit der Folge inhärenter Ungenauigkeiten. Aus diesem Grund sind die Ergebnisse der Umfrage und deren abschließende Aussagekraft in Bezug auf die Inzidenz des SMARD-Syndroms als vorläufiger Schätzwert zu betrachten. Die in der SLR berücksichtigten Quellen beschreiben lediglich eine neu aufgetretene postoperative Diarrhö (62 %). Das SMARD-Syndrom wurde bis heute nicht klar definiert. Aus diesem Grund kann abschließend keine eindeutige Aussage getroffen werden, ob es sich bei den Daten aus der SLR um ein SMARD-Syndrom handelt oder um lediglich eine passagere oder durch eine exokrine PI bedingte Diarrhö. Dennoch spiegelt die SLR die Häufigkeit dieser bis dato mutmaßlich unterschätzen Komplikation wider.

Die Wahrscheinlichkeit, mit der ein SMARD-Syndrom postoperativ auftritt, lässt sich präoperativ bislang nicht abschätzen. Ebenso ist der Verlauf eines SMARD-Syndroms individuell verschieden. In Einzelfällen kann wegen Therapierefraktärität eine deutliche Einschränkung der LQ bestehen bleiben. Hier ist eine umfassende integrative Therapie für den Erhalt der LQ wichtig. Als problematisch sehen wir die bisher noch wenig dokumentierte Anzahl an Patienten mit SMARD-Syndrom, da dadurch klinische Studien erheblich erschwert werden und der Langzeitverlauf schwer beurteilbar ist. Prospektive Studien zur Erfassung der Inzidenz des SMARD-Syndroms, dessen Therapiemöglichkeiten und die damit verbundene Verbesserung der LQ sollten folgen. Insbesondere sehen wir es als sinnvoll an, ein Register zu etablieren bzw. das Syndrom in bestehenden Registern (z. B. der Fachgesellschaft) zu berücksichtigen.

## Fazit für die Praxis

- Das SMARD(„Superior Mesenteric Artery Revascularisation and Dissection-associated diarrhea“)-Syndrom ist ein häufiges und relevantes Krankheitsbild nach Revaskularisation oder Dissektion der A. mesenterica superior bei Pankreasresektionen oder mesenterialen Gefäßeingriffen und kann zu deutlicher Einschränkung der Lebensqualität (LQ) führen.
- Es ist wichtig, das Krankheitsbild zu erkennen und der Schwere der Symptomatik entsprechend zu therapieren, um einer Minderung der LQ entgegenzuwirken. Dabei spielen vor allem Ernährungsberatung, Volumen- und Elektrolytausgleich und eine medikamentöse Therapie der Diarrhö eine Rolle.
- Neuere Therapieansätze sollten in der Praxis v. a. bei therapierefraktären Verläufen erprobt werden.
